# Hydrophobin gene deletion and environmental growth conditions impact mechanical properties of mycelium by affecting the density of the material

**DOI:** 10.1038/s41598-018-23171-2

**Published:** 2018-03-16

**Authors:** Freek V. W. Appels, Jan Dijksterhuis, Catherine E. Lukasiewicz, Kaspar M. B. Jansen, Han A. B. Wösten, Pauline Krijgsheld

**Affiliations:** 1 Microbiology, Department of Biology, Utrecht University, Padualaan 8, 3584 CH Utrecht, The Netherlands; 2 Westerdijk Fungal Biodiversity Institute, Uppsalalaan 8, 3584 CT Utrecht, The Netherlands; 3 Emerging Materials, Department of Design Engineering, Delft University of Technology, Landbergstraat 15, 2628 CE Delft, The Netherlands

## Abstract

Filamentous fungi colonize substrates by forming a mycelium. This network of hyphae can be used as a bio-based material. Here, we assessed the impact of environmental growth conditions and deletion of the hydrophobin gene *sc3* on material properties of the mycelium of the mushroom forming fungus *Schizophyllum commune*. Thermogravimetric analysis showed that Δ*sc3* mycelium retained more water with increasing temperature when compared to the wild type. The Young’s modulus (E) of the mycelium ranged between 438 and 913 MPa when the wild type strain was grown in the dark or in the light at low or high CO_2_ levels. This was accompanied by a maximum tensile strength (σ) of 5.1–9.6 MPa. In contrast, E and σ of the Δ*sc3* strain were 3–4- fold higher with values of 1237–2727 MPa and 15.6–40.4 MPa, respectively. These values correlated with mycelium density, while no differences in chemical composition of the mycelia were observed as shown by ATR-FTIR. Together, genetic modification and environmental growth conditions impact mechanical properties of the mycelium by affecting the density of the mycelium. As a result, mechanical properties of wild type mycelium were similar to those of natural materials, while those of Δ*sc3* were more similar to thermoplastics.

## Introduction

The use of bio-based materials is part of the conversion to a circular economy. These materials are derived from molecules or structures of microbes, plants, macro-algae, and animals. Plant-derived thermoplastic starch^1^, bacterial-derived polyhydroxyalkanoic acid^2^, and fungal mycelium^3–6^ are examples of bio-based materials. Fungal mycelia consist of hyphae that grow at their tips and branch subapically. The internal turgor pressure and the rigid cell walls enable hyphae to penetrate organic material such as plant waste. Secreted enzymes degrade polymers in the substrate into molecules that can be taken up to serve as nutrients. Fungal mycelia can cover huge areas, in particular those of mushroom forming fungi. For instance, the mycelium of an *Armillaria* individual had colonized almost 10 km^2^ of forest^7^.

*Schizophyllum commune* is a model for mushroom forming fungi^8^. Germination of its basidiospores results in a vegetative mycelium that colonizes fallen branches and logs of hardwood. Fusion of two individuals with compatible mating type loci results in a fertile mycelium. Fruiting is induced upon exposure to blue light and ambient CO_2_ (i.e. 400 ppm). The outer layer of the *S. commune* cell wall consists of a water-soluble mucilage of (1,3)(1,6)-β-glucan^9^. This so called schizophyllan is also secreted into the culture medium. A (1,3)-α-linked glucan is located beneath the mucilage, while the inner layer of the cell wall consists of chitin cross-linked to a highly branched (1,3)(1,6)-β-glucan^9,10^. The SC3 hydrophobin impacts cell wall composition of *S. commune*. In the absence of this cell wall protein the amount of schizophyllan is increased, while the amount of glucan that is cross-linked to chitin is reduced^11^. SC3 also attaches hyphae to hydrophobic surfaces^12^, mediates escape of hyphae from the aqueous environment into the air^13^, and makes aerial structures hydrophobic^14,15^. The latter is illustrated by the fact that wild type mycelium has a water contact angle of 115 degrees, being similar to the highly hydrophobic surface of Teflon, while water immediately soaks into the mycelium of the Δ*sc3* strain^15^.

Here, we assessed properties of wild type and Δ*sc3* mycelium of *S. commune* grown in the light or in the dark at 400 or 70,000 ppm CO_2_. Mycelium of strain Δ*sc3* retained more water with increasing temperature when compared to that of the wild type. Both the absence of SC3 and environmental conditions affected mechanical properties of the mycelium, which can be explained by changes in the density in the mycelium. Together, genetic modification and environmental growth conditions can be used to create a palette of mycelium materials.

## Results and Discussion

### Environmental conditions and deletion of *sc3* impact the density of mycelium

**Figure 1:**
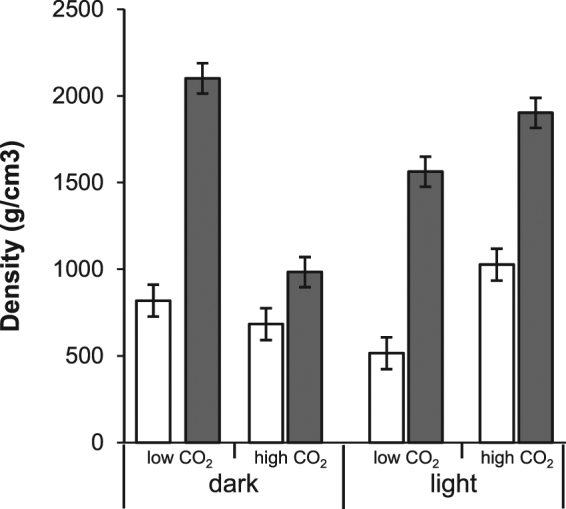
Density of mycelia of liquid static cultures of wild type (non-shaded bars) and *∆sc3* (grey shaded bars) grown in the dark or light at 400 or 70,000 ppm CO_2_. Different letters indicate significant differences (two-tailed independent-sample *t*-test, n = 15, p < 0.05).

Mycelium of the wild type and the hydrophobin deletion strain Δ*sc3* was grown as a liquid static culture (see Material and Methods) in the light or in the dark at 400 ppm (low) or 70,000 ppm (high) CO_2_. Density of wild type mycelium grown at low CO_2_ in the dark or at high CO_2_ in the light was similar (819–1026 kg m^−3^; Fig. 1, Table 1). Lower densities were obtained after growing at high CO_2_ in the dark (683 kg m^−3^) and at low CO_2_ in the light (515 kg m^−3^). The relation between mycelium density, CO_2_, and light is not clear yet. Possibly, it results from interacting signaling pathways that respond to light and CO_2_.

**Table 1:** Young’s modulus (E), maximum tensile strength (σ), elongation at breaking (ε), and density of mycelium of wild type and ∆*sc3 S. commune* strains grown in the light or in the dark at 400 or 70,000 ppm CO_2_. n = 5; average ± SEM.

Strain	Growth condition	E (mPa)	σ (mPa)	ε (%)	Density (kg m^−3^)
Wild type	Dark, low CO_2_	749 ± 49	9.6 ± 1.1	1.2 ± 0.1	819 ± 18
	Dark, high CO_2_	550 ± 48	6.5 ± 0.3	1.4 ± 0.1	683 ± 17
	Light, low CO_2_	438 ± 40	5.1 ± 0.4	1.3 ± 0.1	515 ± 10
	Light, high CO_2_	913 ± 69	9.5 ± 0.6	1.3 ± 0.1	1026 ± 46
∆*sc3*	Dark, low CO_2_	2523 ± 184	33.9 ± 2.8	1.7 ± 0.2	2101 ± 18
	Dark, high CO_2_	1237 ± 108	15.6 ± 2.3	1.9 ± 0.1	984 ± 36
	Light, low CO_2_	1914 ± 156	22.3 ± 1.5	1.8 ± 0.1	1562 ± 56
	Light, high CO_2_	2727 ± 95	40.4 ± 2.5	2.6 ± 0.5	1902 ± 38

**Figure 2:**
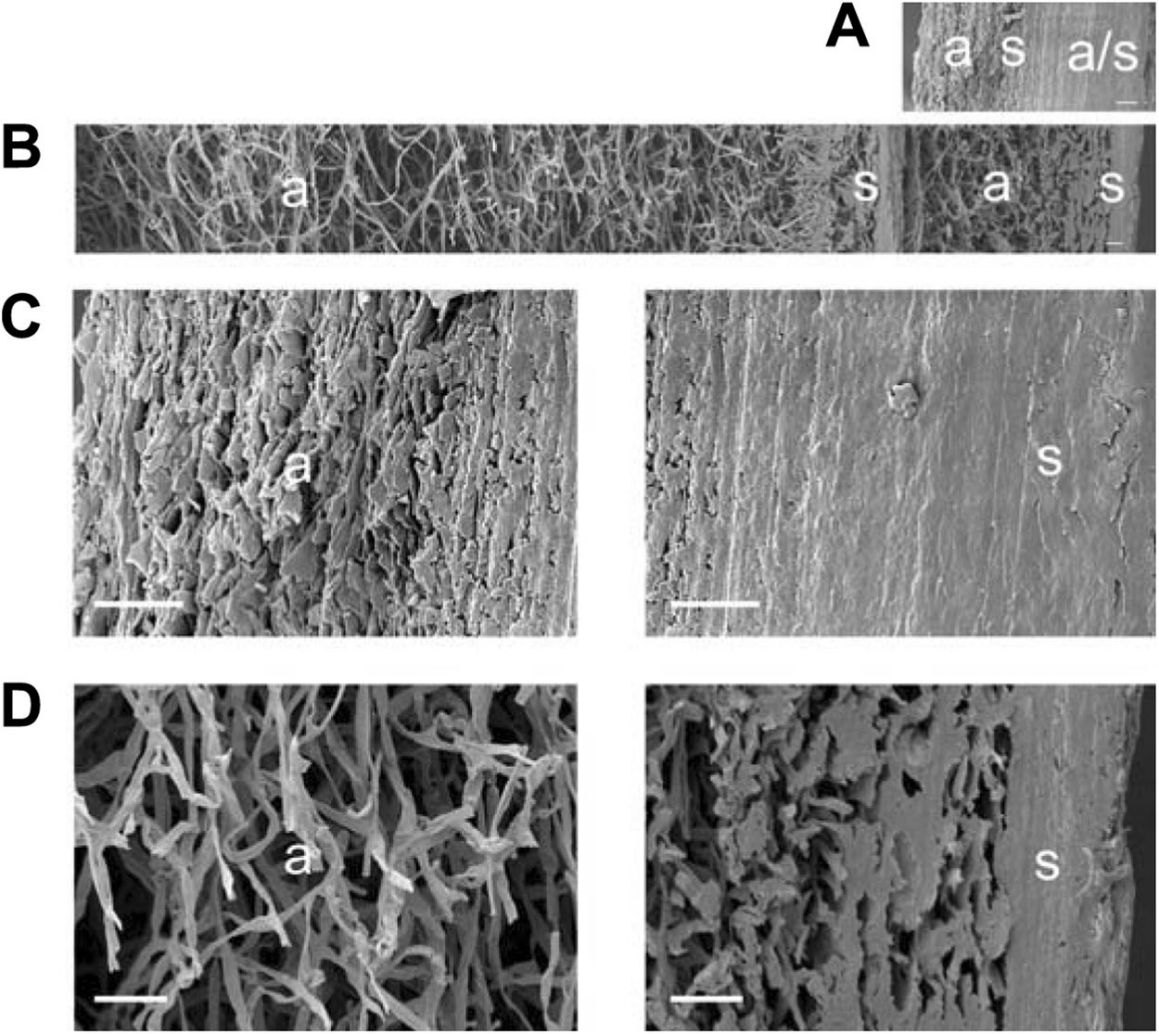
Cryo-SEM microscopy showing an overview (**A,B**) and detailed morphology (**C,D**) of two mycelium layers of *S. commune* Δ*sc3* (**A,C**) and wild type (**B,D**) that had been grown in light and low CO_2_ and that had been dried on top of each other (this was required to enable mechanical analysis of strain Δ*sc3*). a indicates air exposed sites of the mycelium during culturing, while s indicates substrate exposed sites. Bar represents 10 μm.

Density of Δ*sc3* mycelium resulting from the different growth conditions followed a similar trend as the wild type, but was in all conditions 1.4–3 fold higher (Fig. 1; Table 1). As expected^13^, scanning electron microscopy revealed that Δ*sc3* mycelium lacked the thick layer of aerial hyphae observed in the wild type that is characterized by a low hyphal density (Fig. 2). Lower density of the wild type mycelium is also explained by the fact that hyphae of Δ*sc3*, but not those of wild type, were embedded in a mucilage. This mucilage is most probably schizophyllan that is abundantly released by strain Δ*sc3*^11^.

### Environmental conditions and deletion of *sc3* impact mechanical properties of mycelium

**Figure 3:**
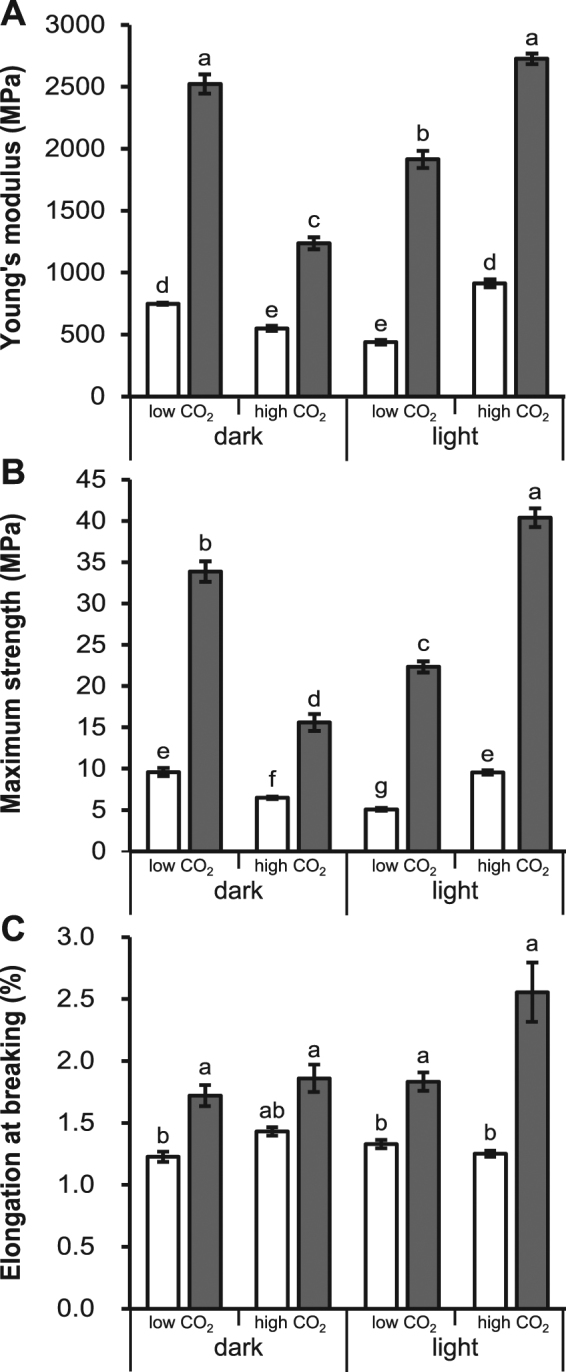
Young’s modulus (**A**), maximum tensile strength at breaking (**B**), and elongation at breaking (**C**) of mycelia of liquid static cultures of wild type (non-shaded bars) and *∆sc3* (grey shaded bars) grown in the dark or light at high or low CO_2_. Different letters indicate significant differences (two-tailed independent sample *t*-test, n = 15, p < 0.05).

Mechanical properties were determined of wild type and Δ*sc3* mycelium grown in liquid static cultures in the light or in the dark at high or low CO_2_. The Young’s modulus (E) of the wild type strain grown in the light increased from 438 to 913 MPa as a result of increased CO_2_ levels (Fig. 3A, Table 1). In contrast, high CO_2_ in the dark resulted in a lower E when compared to low CO_2_ levels (550 to 749 MPa). Similar results were obtained with the maximum strength (Fig. 3B, Table 1). Mycelium of the wild type grown in the dark at high and low CO_2_ had a maximum strength (σ) of 6.5 and 9.6 MPa, respectively. These values were 9.5 and 5.1 MPa, respectively, when wild type mycelium was grown in the light. The elongation at breaking of wild type mycelia was between 1.2 and 1.4% and was not affected by the growth conditions (Fig. 3C; Table 1). Mycelium of Δ*sc3* had a higher E and σ when compared to wild type under all growth conditions (Fig. 3AB; Table 1). E of Δ*sc3* grown in the light increased from 1914 MPa to 2727 MPa as a result of increased CO_2_ levels (Fig. 3A; Table 1). In contrast, an increase in CO_2_ in the dark resulted in a decrease of the Young’s modulus from 2523 MPa to 1237 MPa. Similar results were obtained with σ (Fig. 3B; Table 1). Mycelium of Δ*sc3* grown in the dark at high CO_2_ had a σ of 15.6 MPa, while mycelium grown at low CO_2_ had a σ of 33.9 MPa. These values were 40.4 and 22.3 MPa, respectively, when mycelium was grown in the light. Elongation of Δ*sc3* mycelia at breaking was not affected by the environmental conditions (Fig. 3C; Table 1), but it was higher than wild type in dark and low CO_2_ (1.7 vs 1.2%), light and low CO_2_ (1.8 vs 1.3%), and light and high CO_2_

(2.6 vs 1.3%). Reintroducing *sc3* in strain Δ*sc3* restored E, σ, and elongation at breaking to wild type levels (data not shown), showing that the differences in properties between wild type and Δ*sc3* is due to the absence of SC3. Interestingly, E and σ correlated with the density of the mycelium (R^2^ = 0.91 (p < 0.01), R^2^ = 0.83 (p < 0.01)), respectively. This is generally observed with natural composites and polymers^16^. Together, both wild type and Δ*sc3* mycelium behaved as rigid and brittle materials, also indicated by their typical stress strain curves (Supplementary Figure 2). Notably, E and σ of *S. commune* mycelium were up to 227 and 37-fold stronger when compared to *Pleurotus ostreatus* and *Ganoderma lucidum*^4^. However, elongation at breaking was higher for the latter species being up to 13-fold in the case of *G. lucidum*.

### Mycelia of wild type and ∆*sc3* have a similar chemical composition as indicated by ATR-FTIR

**Figure 4:**
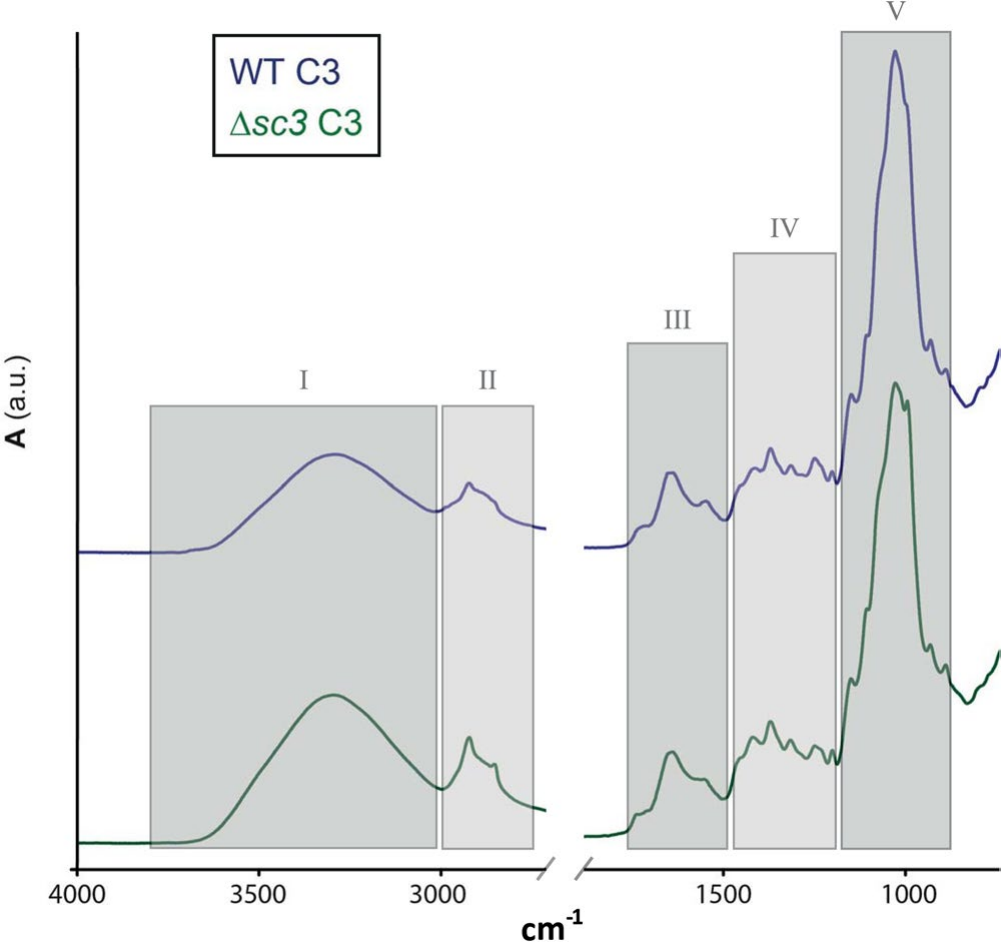
ATR-FTIR spectra of liquid static cultures of *S. commune* wild type and Δ*sc3* grown in light and low CO_2_. Similar absorption peaks were observed between strains, indicating that the mycelia had a similar chemical composition. Definition of wavenumber regions I-V can be found in Table 2. Spectra were obtained in quadruple.

Chemical composition of the mycelia was determined with attenuated total reflectance Fourier-transform infrared spectroscopy (ATR-FTIR). Spectra of wild type and ∆*sc3* were similar and also did not change with the different environmental conditions (Fig. 4; Supplementary Figure 1; Table 2; Supplementary Table S1). As expected^9^, signals originating from lipids and protein were low, while those of carbohydrate were high. Previously, it was shown that ∆*sc3* has an increased amount of schizophyllan and a reduced amount of glucan cross-linked to chitin^11^. ATR-FTIR spectra indicate that the amount of glucan produced by wild type and ∆*sc3* was similar but that the wild type has a higher degree of cross-linking. These results and the correlation between E and σ with density of the mycelium show that differences in mechanical properties between mycelia of wild type and ∆*sc3* grown at different environmental conditions can be solely explained by the density of the mycelium.

**Table 2:** Wavenumber regions in ATR-FTIR spectral windows and their dominating chemical compounds (linked macromolecules), and functional group assignments. Adapted from Naumann^21^.

Symbol	ṽ (cm^−1^)	Macromolecule	Assignment
I	3700–2996	various	O-H, N-H
II	2996–2800	lipid	C-H
III	1800–1485	protein	Amide I + II
IV	1485–1185	Protein, lipid, phosphate compound	CH_2,_ CH_3_ P=O
V	1185–900	Polysaccharide	C-O-C, C-O-P

### Thermogravimetric analysis of mycelial films

**Figure 5:**
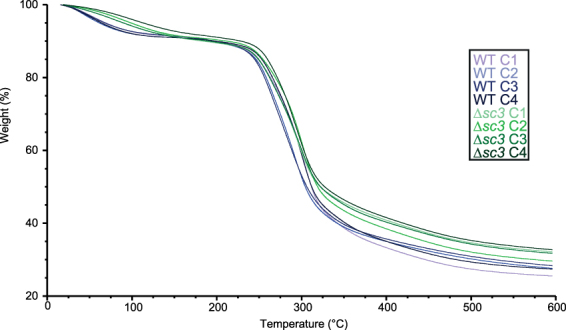
Thermogravimetric analysis of mycelia of *S. commune* wild type and ∆*sc3* grown in the dark at low (C1) and high (C2) CO_2_ and in the light at low (C3) and high (C4) CO_2_. Experiments were performed in duplicate.

Thermogravimetric analysis revealed that thermal degradation of wild type and ∆*sc3* was most pronounced between ± 225 and 300 °C (Fig. 5), being similar to *P. ostreatus* and *G. lucidum*^4^. Notably, weight loss at 100 °C differed between wild type and ∆*sc3* irrespective of the growth conditions (7.5% vs 5.0%, respectively, p < 0.001, data not shown). This indicates that the absence of SC3 leads to a higher water activity. This is in line with the hydrophilicity of the mycelium of the deletion strain, which is in contrast with the hydrophobic nature of the wild-type hyphae that are in contact with air^14,15^.

## Conclusions

Fungal mycelium offers an attractive bio-based material because of its mechanical properties and by the fact that it grows on low quality waste streams such as straw and sawdust. We here showed that mechanical properties of mycelium of *S. commune* can be changed by adapting environmental conditions and by inactivating the *sc3* hydrophobin gene. E and σ of the deletion strain were 3–4-fold higher when compared to the wild type. Notably, they were up to 227- and 37-fold higher, respectively, when compared to mycelium of *P. ostreatus* and *G. lucidum*. Yet, the latter species formed a more elastic mycelium^4^. Both CO_2_ and light affected material properties of *S. commune*. For instance, wild type mycelium showed a 1.7-fold higher E in light compared to dark when grown at high CO_2_. Conversely, E was 2.1-fold higher when light-grown wild type was cultivated at low and high CO_2_. Differences in E and σ between the different strains and culture conditions were shown to be caused by differences in density of the mycelium and not by differences in chemical composition.

**Figure 6:**
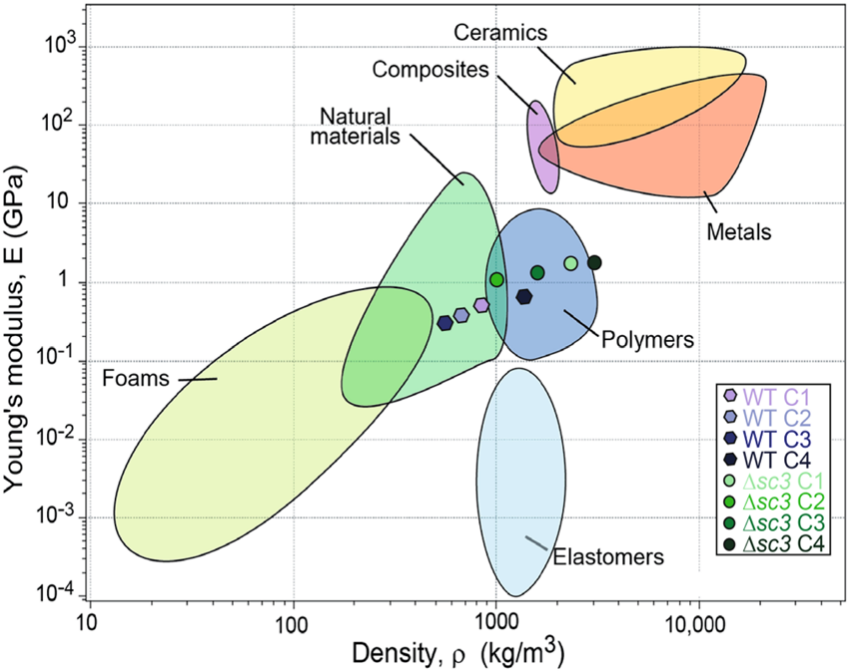
Material family chart of the Young’s modulus (E) (GPa) vs density (kg m^−3^). Mycelium of wild type *S. commune* has properties similar to those of natural materials, while ∆*sc3* mycelium behaves like polymers. Figure adapted from^16^.

The mechanical properties of wild type are similar to those of plant materials (e.g. wood, cork, bamboo) and animal material (leather), while those of Δ*sc3* are more similar to thermoplastics (e.g. polyethylene, polypropylene, polyvinyl chloride). This is mainly caused by increased density and not by increased strength of the material (Fig. 6). The palette of fungal materials can be further diversified by treating mycelium chemically or physically.

## Methods

### Strains and culture conditions

*S. commune* wild type strain 4–39 (CBS 341.81) and its derivative Δ*sc3*^17^ were used in this study. A quarter of a 7-day-old colony grown on agar minimal medium (MM)^18^ was homogenized in 50 ml MM for 30 s at low speed using a Waring Blender (Waring Laboratory, Torrington, England). The homogenate was grown for 24 h at 200 rpm and 30 °C, after which the culture was homogenized. Static liquid cultures were inoculated by taking up 600 mg wet weight mycelial homogenate in a volume of 6 mL MM and spreading it in a 9 cm Petri dish. Cultures were grown at 30 °C in the light (2000 Lux from 5 W LED spot lights, Calex, Rotterdam, the Netherlands) or in the dark at 400 or 70,000 ppm CO_2_. After 3 days, 30 mL MM was applied underneath the mycelial mat^19^ and growth was prolonged for 5 days at 30 °C.

### Complementation of the Δ*sc3* strain

The coding sequence of *sc3* with 995 bp upstream and 301 bp downstream flanking sequences was amplified by PCR using High-Fidelity Phusion polymerase (NEB, Ipswich, USA). The PCR fragment was cloned into plasmid pUC20Nour that consists of a nourseothricin resistance cassette in a pUC20 backbone^20^. The resulting vector pUC20sc3Nour was introduced into Δ*sc3* as described^20^. A first selection was performed using 8 µg mL^−1^ nourseothricin (Jena Biosciences, Jena, Germany) and 500 µg mL^−1^ caffeine (Sigma, St Louis, MO, USA). Nourseothricin resistant colonies were transferred to a 2^nd^ selection plate containing 20 µg mL^−1^ nourseothricin. Colonies were screened by immunodetection using SC3-antiserum^15^. To this end, transformants were grown for 3 days at 30 °C on perforated polycarbonate (PC) membranes (Maine Manufacturing, Sanford, ME, USA; pore size 0.1 µm) on MM agar. The colonies with the underlying PC membrane were transferred for 1 h to a fresh MM agar plate on which a PVDF membrane was placed. Immunodetection of SC3 on the PVDF membranes was performed as described^15^.

### Tensile measurements

Mycelium of two liquid static cultures was dried on top of each other at room temperature. Thickness of the mycelium was measured by a high accuracy length gauge (Heidenhain MT1281, Traunreut, Germany). Tensile measurements of 3 mycelium rectangular specimen (18 × 4 mm) of 5 biological replicas were performed using the Dynamic Mechanical Analyzer Q800 (TA Instruments, New Castle, DE, USA) equipped with an 18 N capacity load cell. The Young’s modulus (E) was obtained by taking the stress/strain slope in the 0.10% to 0.15% strain range. The maximum strength (σ) was obtained from force per unit area, while elongation at breaking point (ε) was obtained by determining the strain (in mm) at the moment of breaking.

### Thermogravimetric analysis

Thermogravimetric analysis was performed with a TGA Q50 (TA Instruments, New Castle, DE, USA). Measurements were performed with 25–30 mg of mycelium in a platinum pan under a constant flow of nitrogen gas (60 mL min^−1^). Temperature increased from 20 to 600 °C with a rate of 10 °C min^−1^. Each experiment was performed using biological duplicates.

### Chemical analysis of mycelial films with ATR-FTIR spectroscopy

Spectra of mycelia were recorded using a PerkinElmer ATR-FTIR spectrometer with a diamond/ZnSe crystal (PerkinElmer, Waltham, MA, USA). Each spectrum was measured between 4000 cm^−1^ to 650 cm^−1^ and compiled from 10 accumulated scans. Four samples were measured per strain per growth condition. Mycelium was placed with their bottom side facing the crystal.

### Scanning Electron microscopy

Mycelium of two liquid static cultures was dried on top of each other at room temperature. Small rectangles (3 × 3 mm) were cut with a scalpel and attached with a 2 mm piece of Scotch tape in a 1 cm ø copper cup. After snap-freezing with liquid nitrogen, samples were transferred to a JEOL 5600 LV scanning electron microscope (JEOL, Tokyo, Japan) by the use of an Oxford CT1500 Cryostation. Ice was removed from the sample by sublimation at −85 °C. Gold was sputter coated for 2 min, after which micrographs were acquired at an acceleration voltage of 5 kV.

### Statistical analysis

Statistical analysis was performed with the software package IBM SPSS statistics 22.0 (IBM Corporation, Armonk, New York) using two-tailed independent-samples *t*-Tests and Pearson correlation analysis (p ≤ 0.05).

## Electronic supplementary material

Supplementary Information
