# Supplementary material for: Hydrophobin gene deletion and environmental growth conditions impact mechanical properties of mycelium by affecting the density of the material
Source: Sci Rep. 2018 Mar 16;8:4703. doi: 10.1038/s41598-018-23171-2 (PMC5856774; doi:10.1038/s41598-018-23171-2)
Supplement: Supplementary file 1 — Supplementary Information [file 41598_2018_23171_MOESM1_ESM.docx]

**Hydrophobin gene deletion and environmental growth conditions impact mechanical properties of mycelium by affecting the density of the material**

Freek VW Appels^1^, Jan Dijksterhuis^2^, Catherine E Lukasiewicz^1^, Kaspar MB Jansen^3^, Han AB Wösten^1^*, and Pauline Krijgsheld^1^.

**Supplementary Figure S1**. ATR-FTIR spectra of wild type and *∆sc3* grown in the dark at low (1,5) and high (2,6) CO_2_ and in the light at low (3,7) and high (4,8) CO_2_.


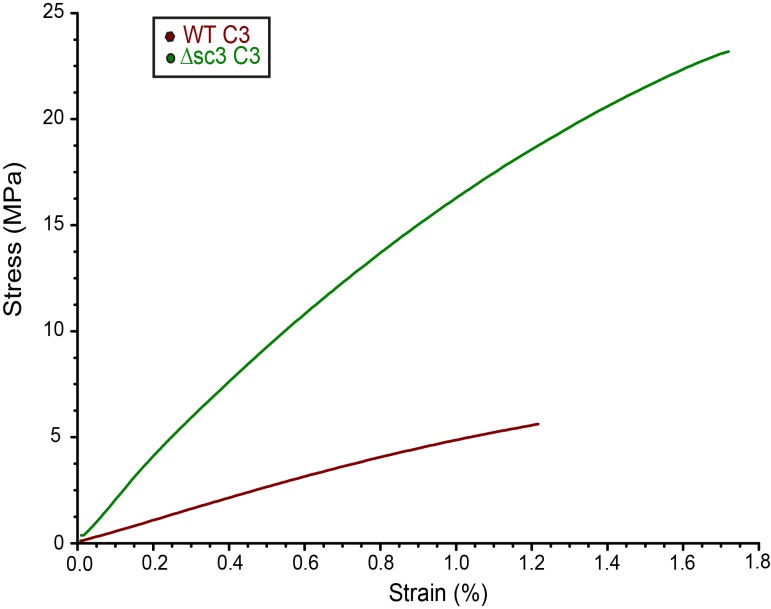


**Supplementary Figure S2**. Examples of stress / strain curves from wild type (red) and *∆sc3* (green) grown in light and low CO_2_.

**Supplementary Table S1**. Overview of some of the peaks obtained with ATR-FTIR for wild type and *∆sc3* mycelial mats grown in light and low CO_2_. Shown are wavenumbers (cm^-1^) with related assignments.

| Assignment | Wavenumbers (cm^-1^) | | Macromolecule | Reference. |
| --- | --- | --- | --- | --- |
|  | wild type | ∆sc3 |  |  |
| Glucan β-anomer C-H bending | 890 | 889 | Polysaccharide | ^1^ |
| Glucan α-anormer C-H deformation | 931 | 932 | Polysaccharide | ^2^ |
| C-C stretching | 1027 | 1027 | Polysaccharide | ^1^ |
| C-O•••H stretching | 1147 | 1148 | Polysaccharide | ^1^ |
| C-C stretching + C-O stretching + C-H deformation | 1202 | 1201 | Polysaccharide | ^2^ |
| PO^2-^ asymmetric stretching | 1249 | 1249 | Nucleic acids | ^1^ |
| C-H bending | 1370 | 1370 | chitin | ^1^ |
| Amide II | 1550 | 1550 | proteins | ^1^ |
| Amide I | 1638 | 1639 | proteins | ^1^ |
| CH_2_ symmetric stretching | 2853 | 2856 | lipids | ^1^ |
| CH_2_ asymmetric stretching | 2923 | 2922 | lipids | ^1^ |
| O-H stretching | 3291 | 3294 | various | ^3^ |

1. Haneef, M., Ceseracciu, L., Canale, C., Bayer, I. S., Heredia-Guerrero, J. A. & Athanassiou, A. Advanced Materials From Fungal Mycelium: Fabrication and Tuning of Physical Properties. *Sci. rep.* **7**, 41292 (2017).
2. Mohaček-Grošev, V., Božac, R, & Puppels, G. J. Vibrational spectroscopic characterization of wild growing mushrooms and toadstools. *Spectrochim. Acta A*, **57**, 2815-2829 (2001).
3. Naumann, A. A novel procedure for strain classification of fungal mycelium by cluster and artificial neural network analysis of Fourier transform infrared (FTIR) spectra. *Analyst*, **134**, 1215-1223 (2009).
